# Case report: Transcatheter edge-to-edge repair after prior surgical tricuspid annuloplasty

**DOI:** 10.3389/fcvm.2022.1044410

**Published:** 2022-10-28

**Authors:** Shazia Afzal, Jafer Haschemi, Florian Bönner, Malte Kelm, Patrick Horn

**Affiliations:** Division of Cardiology, Pulmonology and Vascular Medicine, Medical Faculty, University Düsseldorf, Dūsseldorf, Germany

**Keywords:** TriClip, tricuspid regurgitation, annuloplasty, surgery, percutaneous

## Abstract

Residual and recurrent tricuspid regurgitation may occur frequently after surgical tricuspid valve repair. However, reoperation for tricuspid regurgitation is rare, although many patients are again highly symptomatic. Tricuspid transcatheter edge-to-edge repair (TEER) is a promising therapy for severe tricuspid regurgitation. Herein, we report a 77-year-old woman with recurrent symptomatic massive tricuspid regurgitation 2 years after sutured annuloplasty of the tricuspid valve. TEER was successfully performed using the TriClip^®^ device and tricuspid regurgitation was reduced to a mild degree. In conclusion, tricuspid TEER is feasible following surgical suture annuloplasty. TEER is an alternative option for patients with a failed previous annuloplasty repair for tricuspid regurgitation to undergo a less invasive treatment rather than a potentially higher-risk reoperation.

## Introduction

Residual and recurrent tricuspid regurgitation may occur in 14–30% of patients early after undergoing surgery for all types of tricuspid annuloplasty (1, 2). However, reoperation for tricuspid regurgitation is rare, although many patients are symptomatic (1). The low rate of reoperation may be partly due to the fact that reoperation to repair or replace the regurgitant tricuspid valve is a high-risk procedure in high-risk patients. Transcatheter edge-to edge repair (TEER) is a promising therapy for severe tricuspid regurgitation (3, 4). Up to now, two TEER devices have CE mark for the treatment of tricuspid regurgitation, the TriClip^®^ (Abbott, Santa Clara, CA, USA) and the PASCAL^®^ Implant System (Edwards Lifesciences, Irvine, CA, USA). We here describe a case of a patient who was treated with the TriClip^®^ device for recurrent tricuspid regurgitation after previous tricuspid annuloplasty.

## Case presentation

The patient was 77-year-old woman with a history of ischemic cardiomyopathy and severe functional mitral and tricuspid regurgitation. Two years prior, she had undergone coronary bypass surgery, mitral valve ring annuloplasty, and suture annuloplasty of the tricuspid valve (DeVega).

The patient complained of worsening heart failure symptoms, particularly peripheral edema, decreased physical capacity, and shortness of breath (New York Heart Association (NYHA) Functional Class III). Upon admission, physical examination revealed marked peripheral edema, jugular venous distension, and irregular heartbeat. The electrocardiogram showed atrial fibrillation. NT-proB-type natriuretic peptide level was 2,270 pg/mL.

Echocardiography demonstrated normal left ventricular function and mild mitral regurgitation after mitral ring annuloplasty but massive tricuspid regurgitation (vena contractae width of 15 mm) due to gradual dilation of the tricuspid annulus (diameter of 40 mm) (Supplementary Video S1). The patient was evaluated as to be at high risk for re-surgery of the tricuspid valve (EuroScore II 4.8%) and was considered for TTVr by the heart team.

**Figure 1:**
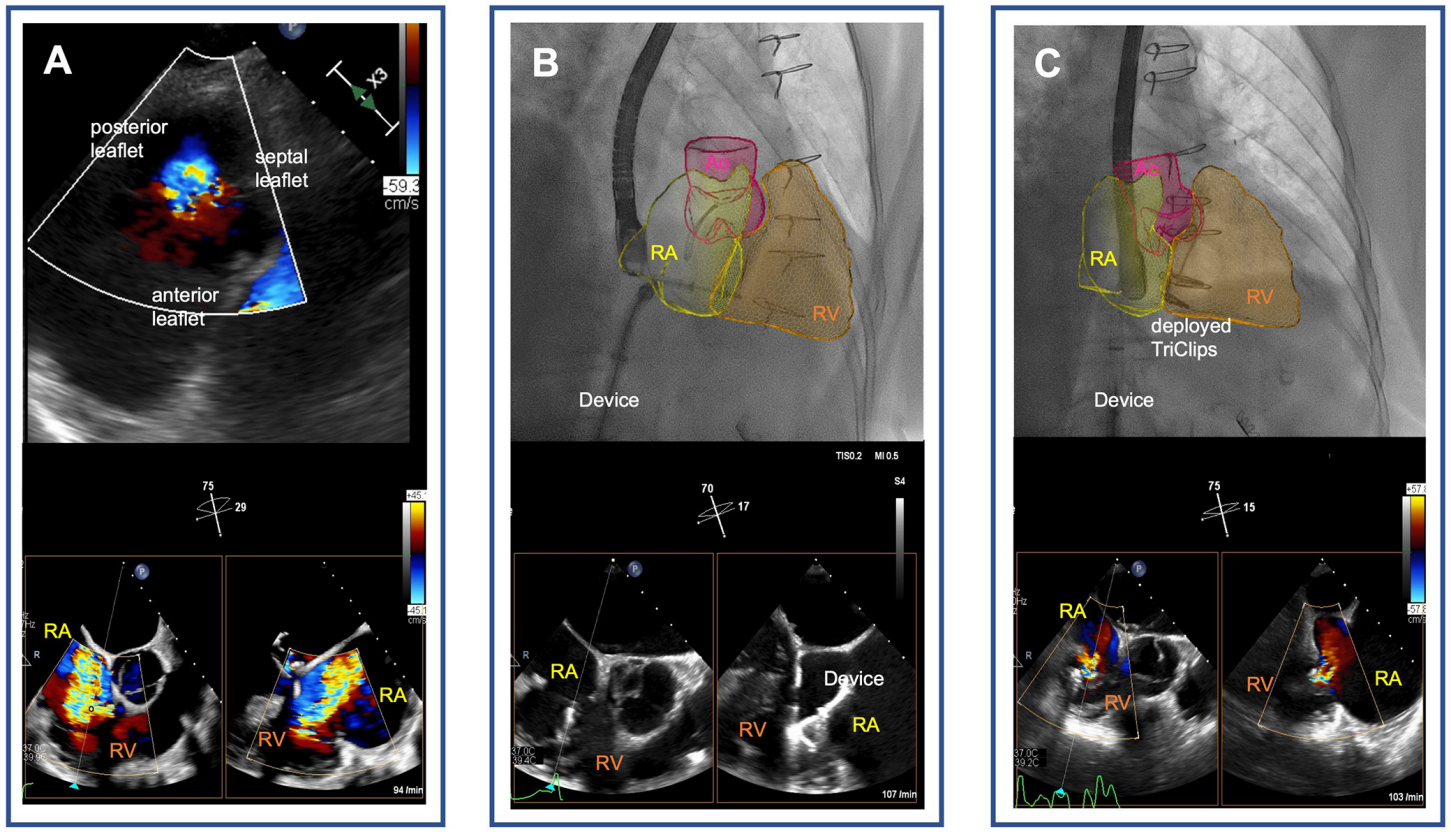
Tricuspid transcatheter edge-to-edge repair following surgical tricuspid annuloplasty. **(A)** Severe functional tricuspid regurgitation following sutured annuloplasty [transgastric view on the tricuspid valve **(upper panel)** and biplane transesophageal echocardiographic images of the right ventricular outflow tract **(lower panel)**]. **(B)** Real-time fusion imaging-assisted navigation of the transcatheter tricuspid valve repair device through the right atrium pointing to the tricuspid annulus **(upper panel)**. Biplane transesophageal echocardiographic images of the right ventricular outflow tract showing grasping of the septal and anterior leaflets of the tricuspid valve with the TriClip device **(lower panel)**. **(C)** Mild tricuspid regurgitation after deployment of two TriClips [real-time fusion of echocardiography-derived right heart cavities on fluoroscopy visualizes the TriClips **(upper panel)**, and biplane esophageal echocardiographic image demonstrates reduction of tricuspid regurgitation to mild degree **(lower panel)**]. RA, right atrium; RV, right ventricle; Ao, aortae.

TEER was performed by using the TriClip^®^ device (Abbott, Vascular GmbH). During the TEER procedure, it was difficult to visualize the septal leaflet of the tricuspid valve in transesophageal echocardiography (TEE) due to shadowing from the annuloplasty ring on the mitral site. This was resolved by utilizing an atypically higher degree of probe rotation (Supplementary Video S2). Previous sutured annuloplasty of the tricuspid valve did not affect imaging of the valve or the procedure itself. The first device was used to grasp the anterior and septal leaflets. The second device was deployed to grasp the posterior and septal leaflets. Tricuspid regurgitation was reduced to a mild degree in postprocedural TEE (Figure 1), and in transthoracic echocardiography that was performed at the time of discharge (Supplementary Video S3). Hemodynamic improvement with slightly reduced left atrial pressure (from 12 mmHg before the procedure to 9 mmHg after the procedure) and markedly increased cardiac index (from 1.8 to 2.5 L·min^−1^·m^−2^). After 1 month of follow-up, repeat echocardiography demonstrated tricuspid regurgitation grade 1 without stenosis (transvalvular gradient of 1 mmHg). The patient reported that physical capacity and dypnea had improved (to NYHA functional class I).

## Discussion

Residual and recurrent tricuspid regurgitation occurs in 14% of patients early during the first month after operation for all types of tricuspid annuloplasty (1). In non-ring annuloplasty as the DeVega technique, substantial worsening of late tricuspid regurgitation has been reported in up to 30% of all patients, presumably due to gradual redilatation of the annulus (1, 2). However, reoperation for tricuspid regurgitation is rare although many patients belong to NYHA class III or IV (1). The low rate of reoperation may be partly due to the fact that reoperation to repair or replace the regurgitant tricuspid valve is a high-risk procedure in high-risk patients (5).

TEER is an alternative option for patients with a failed previous annuloplasty repair for functional tricuspid regurgitation to undergo less invasive treatment, as demonstrated in our case. TEER following surgical tricuspid valve repair has previously been reported in two cases describing the off-label use of the MitraClip^®^ device (6, 7). In another case report, recurrent TR after ring annuloplasty of the tricuspid valve was successfully treated by using the PASCAL^®^ device (8). To our knowledge, this is the first case to demonstrate that TEER using the TriClip^®^ device is feasible following surgical suture annuloplasty.

However, three issues should be considered before considering TEER in patients after previous tricuspid annuloplasty.

First, we must understand the reason for resistant or recurrent tricuspid regurgitation, and have to assess the chance of successfully treating tricuspid regurgitation. In patients with previous sutured annuloplasty, redilatation of the annulus, tethering of leaflets, or tearing of sutures can be easily addressed with TEER. In patients with previous ring annuloplasty, percutaneous valve-in-ring transcatheter heart valve implantation might be an alternative option to TEER. Cardiac CT measurements are required for pre-procedural planning when other devices are considered beyond TEER (valve-in-ring heart valve implantations or transcatheter tricuspid annuloplasty). Treating a tissue tear close to the annuloplasty ring or ring dehiscence may be challenging or even impossible with TEER.

Second, TEER is inherently dependent on echocardiographic image quality during the procedure, as the TEER device must be guided to the tricuspid valve through the right atrium, appropriately oriented on the tricuspid valve leaflets to optimally reduce tricuspid regurgitation, and adequate leaflet insertion of the leaflets must be determined. A previously placed surgical annuloplasty ring may limit the guidance of device placement and the assessment of leaflet insertion by creating an echocardiographic shadow. In the TRILUMINATE study, 25% of the patients who underwent successful TEER had previous mitral intervention (mainly surgery) (9). Thus, imaging may be challenging but there is no limitation for TEER in patients with prior mitral valve procedure. In the present case, even previous surgery at the tricuspid site was no obstacle for TEER. In patients with prior ring annuloplasty on the tricuspid valve, imaging of the leaflets may be challenging because of shadowing of the ring. To overcome these imaging difficulties, intracardiac echocardiography can be used as an alternative imaging approach (10).

Third, in patients with a previously placed surgical annuloplasty ring, there is a potential for tricuspid stenosis, which may be worsened by the reduction in valve area following TEER device placement. Therefore, assessment of the transvalvular tricuspid valve gradient prior to and during the procedure is mandatory.

In our case, tricuspid regurgitation recurrence after annuloplasty was due to redilatation of the annulus, and imaging quality was adequate despite the annuloplasty ring on the mitral site, the transvalvular gradient across the tricuspid valve, and the risk of stenosis induced by an edge-to-edge repair technique was considered low. Thus, in our patient, recurrent tricuspid regurgitation after a previous surgery could be treated safely and efficiently.

## Conclusion

TEER is feasible following surgical suture annuloplasty of the tricuspid valve. This is an alternative option for patients with a failed previous annuloplasty repair for functional tricuspid regurgitation to undergo a less invasive treatment rather than a potentially higher-risk reoperation to repair or replace the regurgitant tricuspid valve. More data with longer follow-up are needed to confirm these early findings.

## Data availability statement

The original contributions presented in the study are included in the article/Supplementary material, further inquiries can be directed to the corresponding author.

## Ethics statement

Written informed consent was obtained from the individual(s) for the publication of any potentially identifiable images or data included in this article.

## Author contributions

SA, JH, and PH wrote the original manuscript, performed formal analysis, and revised the manuscript and treated the patient involved in this case report. FB and MK were involved in supervision and manuscript review and editing. PH conceptualized the study and responsible for the overall content. All authors contributed to the article and approved the submitted version.

## Conflict of interest

Author PH has received travel support from Abbott, outside the submitted work. The remaining authors declare that the research was conducted in the absence of any commercial or financial relationships that could be construed as a potential conflict of interest.

## Publisher's note

All claims expressed in this article are solely those of the authors and do not necessarily represent those of their affiliated organizations, or those of the publisher, the editors and the reviewers. Any product that may be evaluated in this article, or claim that may be made by its manufacturer, is not guaranteed or endorsed by the publisher.

## Supplementary material

Supplementary Video S1 Transgastric echocardiographic **(first loop)** and transesophageal echocardiographic image **(second loop)** of the tricuspid valve demonstrating severe functional tricuspid regurgitation due to redilatation of the annulus following sutured annuloplasty.

Supplementary Video S2 Transesophageal echocardiographic image demonstrating difficult visualization of the septal leaflet of the tricuspid valve in transesophageal echocardiography (TEE) due to shadowing from the annuloplasty ring on the mitral site.

Supplementary Video S3 Transesophageal echocardiographic image demonstrating grasping of the septal and anterior leaflets of the tricuspid valve using a high degree of probe rotation to overcome shadowing.

Supplementary Video S4 Biplane transesophageal echocardiographic image demonstrating reduction of tricuspid regurgitation to mild degree after deployment of two TriClips.

Supplementary Video S5 Transthoracic echocardiographic image demonstrating mild tricuspid regurgitation assessed at the time of discharge after a successful transcatheter tricuspid valve repair.
